# Increased Risk of Kawasaki Disease in Infants Born of Mothers With Immune Disorders

**DOI:** 10.3389/fped.2021.659598

**Published:** 2021-05-14

**Authors:** Hsiao-Wen Chu, Chien-Heng Lin, Ming-Chih Lin, Ya-Chi Hsu

**Affiliations:** ^1^Children's Medical Center, Taichung Veterans General Hospital, Taichung, Taiwan; ^2^Department of Medical Research, Taichung Veterans General Hospital, Taichung, Taiwan; ^3^School of Medicine, National Yang-Ming University, Taipei, Taiwan; ^4^Department of Food and Nutrition, Providence University, Taichung, Taiwan; ^5^School of Medicine, Chung Shan Medical University, Taichung, Taiwan

**Keywords:** Kawasaki disease, autoimmune disease, allergic disease, maternal child interaction, hereditary

## Abstract

**Introduction:** Genetic susceptibility and immune dysregulation play important roles in the pathogenesis of Kawasaki disease (KD). However, it is still unclear whether KD causes immune disorder later in life or whether inherited susceptibility to immune disorders causes KD. The aim of this study was to elucidate whether inherited immune disease properties from mothers increase the risk of KD from a population-based perspective.

**Method:** Taiwan's National Health Insurance Research Database was the main data source in this study. Parents and children were linked using the Taiwan Maternal and Child Health Database. Patients diagnosed with KD and younger than 18 years from 2004 to 2015 were enrolled as the study population. The control group was randomly selected from individuals without the diagnosis of KD matched by age, index year, sex, and urbanization level at a ratio of 1 to 10. The prevalence of maternal autoimmune and allergic diseases was compared between groups.

**Results:** In total, 7,178 children were found to have been diagnosed with Kawasaki disease. Then 71,780 children matched by index year, gender, and urbanization were randomly selected to serve as the control group. Children born from mothers with asthma and allergic rhinitis had a higher risk of developing KD. Children of mothers with an autoimmune disorder had a significantly increased tendency to develop KD. Maternal numbers of autoimmune disorders showed a dose-dependent relationship with KD incidence.

**Conclusion:** This is the first population-based study to investigate maternal immune diseases and the risk of KD in their children. Children of mothers with immune disorders tend to have a higher risk of KD.

## Introduction

Kawasaki disease (KD) was first described by Dr. Kawasaki in 1967 and is characterized by systemic vasculitis, predominantly in preschool children. It is also the most common acquired pediatric heart disease in most industrialized countries (1–3). The typical features of KD include fever lasting more than 5 days, as well as oral mucosa change, such as strawberry tongue, bilateral conjunctival ingestion, polymorphous skin rashes, desquamation over fingertips, and cervical lymphadenopathy (2–4). One of the most severe complications of KD is coronary artery aneurysm, which may cause acute myocardial infarction, aneurysm rupture, or coronary stenosis (3–7).

Although the pathogenesis of KD is still unclear, genetic susceptibility, and immune dysregulation play important roles (8–10). Cytokines and enzymes which can cause aneurysms are released by the activated innate and adaptive immune system (11, 12). Characteristics of allergic reaction, such as Th2 cytokines, elevated IgE, and eosinophilia, were detected in the peripheral blood of KD patients (13–16). Genetic susceptibility has also been linked to genes associated with the immune system (3, 17, 18). Children with KD have a tendency to develop atopy later in life (19, 20). However, it is still unclear whether KD causes immune disorder later in life or inherited susceptibility toward immune disorders causes KD.

The aim of this study was to elucidate whether inherited immune disease properties in mothers increase the risk of KD from a population-based perspective.

## Methods and Materials

### Database Sources

In 1995, a single-payer National Health Insurance (NHI) program with mandatory enrollment was launched in Taiwan. It covers 99.99% of Taiwan's population. The National Health Insurance Research Database (NHIRD), which contains all of the claims data of NHI beneficiaries, was established for public research in 2002 (21, 22). In 2015, the Ministry of Health and Welfare (MOHW) further integrated the NHIRD with other health-related databases at the Health and Welfare Data Center (HWDC) (21, 23).

The ambulatory care expenditures by visit (CD) files and the inpatient expenditures by admission (DD) files from the NHIRD were the main source of data used for analysis. Parents and children were linked using the Taiwan Maternal and Child Health Database (MCHD), which is overseen by Taiwan's Health Promotion Administration (HPA).

### Study Population and Study Design

**Figure 1:**
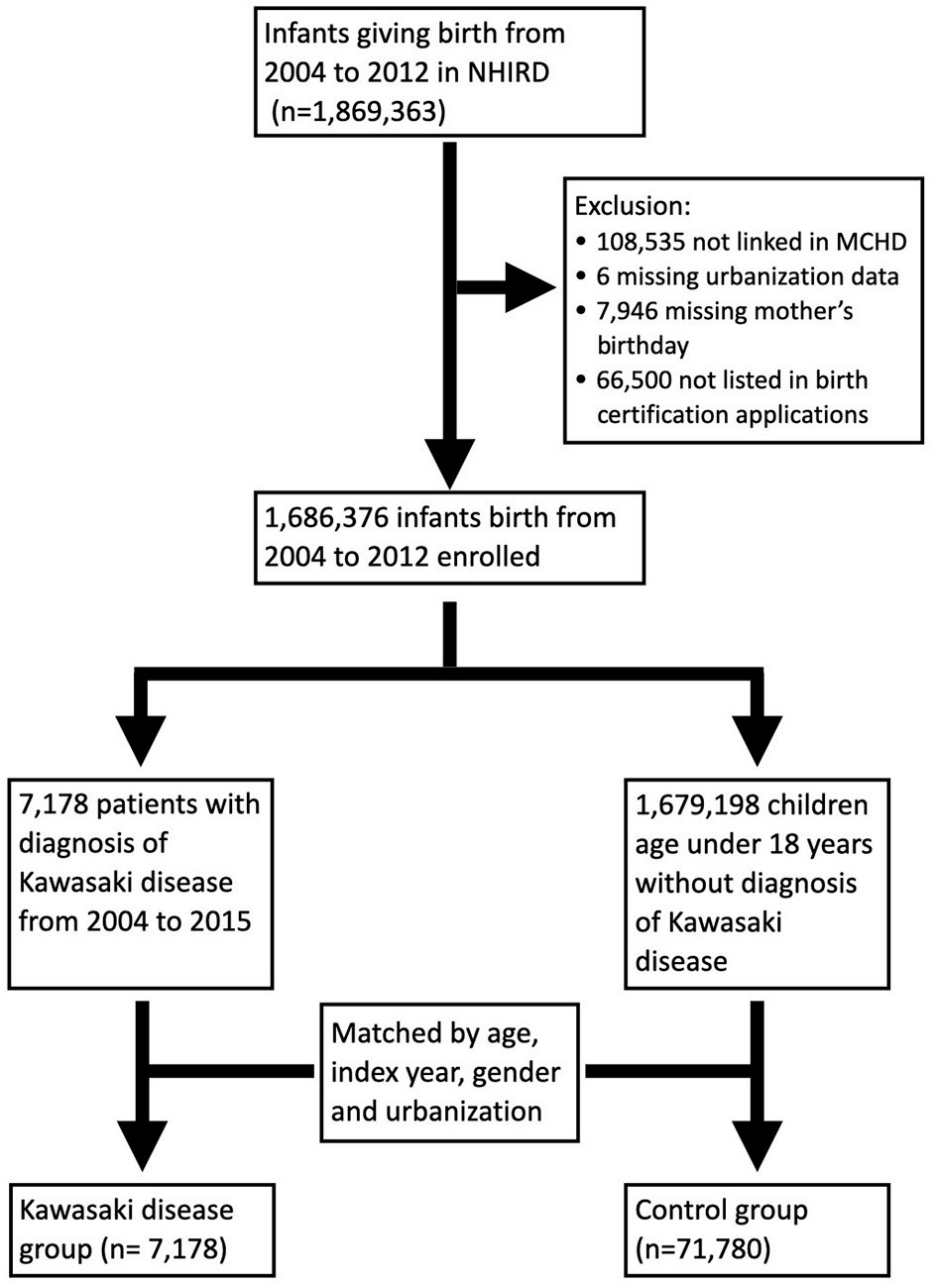
Study population and study design.

Because collection of data related to MCHD began in 2004, infants born between 2004 and 2012 were included in this study. Infants and mothers were linked through the MCHD. Exclusion criteria included individuals with missing values, such as the urbanization level, the mother's birthday, or loss of link in the MCHD. Patients who were diagnosed with KD and aged younger than 18 years from 2004 to 2015 were enrolled as the study population. The control group was selected from individuals without a diagnosis of KD matched by age, index year, sex, and urbanization level at a ratio of 1–10 (Figure 1). Because mothers with autoimmune disease are rare, we tried to increase statistical power by adding more numbers in the control group. The prevalence of maternal autoimmune and allergic diseases including systemic lupus erythematosus (SLE), Sjogren's syndrome (SS), rheumatoid arthritis (RA), bronchial asthma (BA), allergic rhinitis (AR), and atopic dermatitis (AD) were compared between groups. Prematurity and maternal age at pregnancy were listed as cofactors.

The diagnosis of diseases was based on the International Classification of Diseases, 9th revision, Clinical Modification (ICD9-CM). We identified patients with a diagnosis of KD using the main admission code ICD9-CM 446.1. Maternal diseases were defined using the diagnosis codes SLE (ICD9-CM 710.0), SS (ICD9-CM 710.2), RA (ICD9-CM 714.0), BA (ICD9-CM 493), AR (ICD9-CM 477), and AD (ICD9-CM 691) in the records of outpatient visits more than three times or at least one admission before pregnancy. The protocol was approved by the Institutional Review Board of Taichung Veterans General Hospital.

### Statistical Analysis

SAS 9.4 (SAS Institute Inc. Cary, NC, USA) was used for data retrieval and analysis. The mean and standard deviation were used for descriptive statistical analysis of the continuous variables. Number and percentage were used to describe the categorical data. An independent *t*-test was used to compare the continuous variables. A chi-square test was applied for comparing categorical data. A crude odds ratio for developing KD was calculated by univariate logistic regression models. Then an adjusted odds ratio was further calculated by multiple logistic regression models. Odds ratios for KD according to incidence of autoimmune disease were calculated to test whether a dose-dependent effect existed. Because collinearity may exist between asthma and allergic rhinitis, interaction between these two factors in logistic regression models was further tested to determine whether they were independent of each other.

## Results

In total, 1,686,376 infants born between 2004 and 2015 were enrolled in this study. Among them, 7,178 children were diagnosed with KD. A total of 71,780 children were randomly selected from the remaining 1,679,198 children matched by index year, gender, and urbanization at a 1:10 ratio to serve as the control group (Figure 1).

The demographic characteristics of the subjects in the KD and control groups are summarized in Table 1. The average age of KD diagnosis was 1.72 years old (SD = 1.46). Maternal age at pregnancy was older in the KD group than in the control group (29.2 vs. 29.7, *p* < 0.001). There was also a significant difference in gestational age between the KD group and the control group (38.3 vs. 38.2, *p* = 0.01). However, because of large case numbers, such small differences can still reach a statistically significant *p*-value. However, the difference did not make sense clinically.

**Table 1:** Demographic data of patients with KD and the control group.

**Variable**	**KD group**	**Control group**	***p*-value**
	**(*N* = 7,178)**	**(*N* = 71,780)**	
**Child status**			
KD diagnosed age, year (SD)	1.72 (1.46)		
Sex			>0.99
Female	2,767 (38.6%)	27,670 (38.6%)	
Male	4,411 (61.4%)	44,110 (61.4%)	
Urbanization levels			>0.99
1 (highest)	2,225 (31.0%)	22,250 (31.0%)	
2	2,377 (33.1%)	23,770 (33.1%)	
3	1,308 (18.2%)	13,080 (18.2%)	
4 + (lowest)	1,268 (17.7%)	12,680 (17.7%)	
**Mother status**			
Age at pregnancy (SD)	29.7 (4.58)	29.2 (4.77)	<0.001^*^
Gestational age, week (SD)	38.2 (1.68)	38.3 (1.64)	0.01^*^
Birth order			0.66
1	4,867 (67.8%)	48,556 (67.7%)	
2	2,086 (29.1%)	20,829 (29.0%)	
3+	225 (3.13%)	2,395 (3.34%)	
Number of births			0.15
Singleton	6,956 (96.9%)	69,774 (97.2%)	
Multiple	222 (3.09%)	2,006 (2.79%)	

* *t-test, p < 0.05*.

*KD, Kawasaki disease*.

In the univariate model, the maternal age at pregnancy had a significant impact on the occurrence of KD. Children born of mothers with asthma (OR 1.15, 95% CI 1.05, 1.27) or allergic rhinitis (OR 1.09, 95% CI 1.04, 1.15) had a higher risk for developing KD. In the multiple logistic regression model after adjusting for cofactors, maternal asthma and allergic rhinitis were still significant risk factors for KD (Table 2).

**Table 2:** The birth data and maternal diseases of KD patients and control group.

**Variables**	**KD group**	**Control group**	**Crude OR**	**Adjusted OR**
	***N* = 7,178**	***N* = 71,780**	**(95% CI)**	**(95% CI)**
**Prematurity**				
No	6,508 (90.7%)	65,426 (91.2%)	Ref	Ref
Yes	670 (9.33%)	6,354 (8.85%)	1.06 (0.98, 1.15)	1.05 (0.97, 1.14)
**Age at pregnancy**				
<18	48 (0.67%)	715 (1.00%)	Ref	Ref
18–34	6,235 (86.9%)	63,044 (87.8%)	1.47 (1.10, 1.97)	1.47 (1.09, 1.96)
≥35	895 (12.5%)	8,021 (11.2%)	1.66 (1.23, 2.24)	1.65 (1.22, 2.22)
**Autoimmune disease**				
SLE	34 (0.47%)	246 (0.34%)	1.39 (0.97, 1.98)	1.25 (0.87, 1.81)
Sjogren's syndrome	79 (1.10%)	635 (0.88%)	1.25 (0.99, 1.58)	1.16 (0.91, 1.48)
RA	41 (0.57%)	313 (0.44%)	1.31 (0.95, 1.82)	1.22 (0.88, 1.70)
**Allergic disease**				
Asthma	497 (6.92%)	4,352 (6.06%)	1.15 (1.05, 1.27)	1.11 (1.01, 1.23)
Allergic rhinitis	2,224 (31.0%)	20,889 (29.1%)	1.09 (1.04, 1.15)	1.07 (1.02, 1.13)
Atopic dermatitis	374 (5.21%)	3,471 (4.84%)	1.08 (0.97, 1.21)	1.06 (0.95, 1.18)

*Model adjusted for pregnancy, pregnancy age, autoimmune diseases, and allergic diseases*.

*KD, Kawasaki disease*.

With regard to maternal autoimmune diseases, infants born of mothers with an autoimmune disorder had a significant tendency to develop KD either in the univariate or multiple logistic regression models (OR = 1.11, 95% CI 1.06, 1.17). Moreover, maternal numbers of autoimmune disorders showed a dose-dependent relationship with KD incidence (OR = 1.21, 95% CI 1.1, 1.33) (Table 3).

**Table 3:** Odds ratio of acquiring Kawasaki disease for children of mothers with immune disorders.

**Variables**	**KD group**	**Control group**	**Crude OR**	**Adjusted OR**
	***N* = 7,178**	***N* = 71,780**	**(95% CI)**	**(95% CI)**
**Immune disease**				
No	4,530 (63.1%)	47,035 (65.5%)	Ref	Ref
Yes	2,648 (36.9%)	24,745 (34.5%)	1.11 (1.06, 1.17)	1.10 (1.05, 1.16)
**No. of immune diseases**				
None	4,530 (63.1%)	47,035 (65.5%)	Ref	Ref
1	2,106 (29.3%)	20,098 (28.0%)	1.09 (1.03,1.15)	1.08 (1.02, 1.14)
2+	542 (7.55%)	4,647 (6.47%)	1.21 (1.1, 1.33)	1.20 (1.09, 1.32)

*Model adjusted for week, age at pregnancy*.

*KD, Kawasaki disease*.

The interaction between maternal allergic rhinitis and asthma was further analyzed. Their impacts on KD incidence were independent, and the interaction term in the multiple model was not significant either (Table 4).

**Table 4:** Odds ratio of maternal allergic rhinitis and asthma in KD group and control group.

**AR**	**Asthma**	**KD group**	**Control group**	**Crude OR**	**Adjusted OR**
		***N* = 7,178**	***N* = 71,780**	**(95% CI)**	**(95% CI)**
No	No	4,811 (67.0%)	49,515 (69.0%)	Ref	Ref
Yes	No	1,870 (26.0%)	17,913 (25.0%)	1.07 (1.02, 1.14)	1.07 (1.01, 1.13)
No	Yes	143 (1.99%)	1,376 (1.92%)	1.07 (0.90, 1.27)	1.07 (0.90, 1.27)
Yes	Yes	354 (4.93%)	2,976 (4.15%)	1.22 (1.09, 1.37)	1.21 (1.08, 1.36)

*Model adjusted for week, age at pregnancy*.

*KD, Kawasaki disease*.

*p for interaction = 0.37*.

## Discussion

This study investigated the association between maternal immune diseases and the risk of KD occurrence in their children from a nationwide perspective. Maternal asthma and maternal allergic rhinitis were significant risk factors for KD occurrence in their children. Infants born of mothers with autoimmune disorders also tended to have a higher incidence of KD later in life.

Although the pathogenesis of KD is still unclear, the involvement of genetic susceptibility along with an infectious or antigen trigger activating the innate and adaptive immune system has been hypothesized (4, 24, 25). A higher incidence of KD in siblings of patients with KD further supports the notion that susceptibility to KD is heritable (26, 27).

KD has a strong correlation with immune disorders. Elevated serum IgE levels in KD patients imply that a relationship exists between KD and allergic diseases (28). Previous studies also illustrated the activation of the Th1 immune reaction from interferon-Gamma, tumor necrosis factor-alpha, IL-1 and IL-10, as well as Th2-mediated immune reactions, such as IL-4, IL5, and IL-13 during the acute stage of KD (29, 30). With regard to allergic diseases, Kuo et al. performed a population-based study in Taiwan and reported a higher risk of asthma and allergic rhinitis in patients with KD (19). Tsai et al. showed an atopic tendency with allergic rhinitis and asthma in patients with KD from their 1st year of life up to school age (20). Wei et al. reported in a nationwide study that patients with urticaria, allergic rhinitis, or allergic dermatitis had increased risk of KD (31). Webster et al. found that the KD patients had more admissions due to infection or allergic diseases before they developed KD and their relatives also had a greater likelihood of being admitted with asthma, allergy or infection (32). It has also been reported that KD patients and their families had a higher prevalence of atopic dermatitis at preschool age (33, 34). However, whether a tendency toward atopy causes KD or whether KD induces atopy has never been investigated. This study provides initial evidence that the inherited tendency toward atopy may have an impact on the pathogenesis of KD.

Immune complexes have been reported in patients with Kawasaki disease and play an important role in the pathogenesis (9). Autoimmune diseases such as SLE, RA, and Sjogren's syndrome are related to autoantibodies and immune complexes. Zhou et al. reported in a meta-analysis that a sequence similarity 167A-B lymphoid tyrosine kinase (FAM167A-BLK) rs2736340 polymorphism in affected families had a positive correlation with autoimmune diseases as well as KD (35). In our study, numbers of maternal autoimmune disease had a significant and dose-dependent impact on KD occurrence. No specific autoimmune disease reached a statistically significant level, possibly due to the limited case numbers.

This study had certain limitations. The data source was national health insurance claims data. Laboratory data were not included. The disease diagnosis was mainly decided by physicians' coding. The subtypes of KD, such as complete, incomplete, and atypical KD, cannot be classified. Furthermore, data validation cannot be performed because personal identification data are not allowed to be released from the data center. Thus, certain misclassifications may have existed. Several immune diseases, such as systemic sclerosis, dermatomyositis, and polymyositis, were excluded due to insufficient cases and lack of statistical power.

This is the first population-based study to investigate maternal immune diseases and the risk of KD in their children. Children of mothers with immune disorders tended to have a higher risk of KD. Further observational studies or prospective cohort studies in other institutions and countries are needed to verify and corroborate these findings.

## Data Availability Statement

The original contributions presented in the study are included in the article/supplementary materials, further inquiries can be directed to the corresponding author.

## Ethics Statement

The studies involving human participants were reviewed and approved by The Institutional Review Board of Taichung Veterans General Hospital. Written informed consent from the participants' legal guardian/next of kin was not required to participate in this study in accordance with the national legislation and the institutional requirements.

## Author Contributions

M-CL: conceptualization and methodology. C-HL: data curation and formal analysis. H-WC: writing (original draft). Y-CH and M-CL: writing, review, and editing. All authors contributed to the article and approved the submitted version.

## Conflict of Interest

The authors declare that the research was conducted in the absence of any commercial or financial relationships that could be construed as a potential conflict of interest.

## References

[1] Kawasaki T. Acute febrile mucocutaneous syndrome with lymphoid involvement with specific desquamation of the fingers and toes in children. Arerugi. (1967) 16:178–222. 6062087

[2] Kawasaki T Kosaki F Okawa S Shigematsu I Yanagawa H. A new infantile acute febrile mucocutaneous lymph node syndrome (MLNS) prevailing in Japan. Pediatrics. (1974) 54:271–6. 4153258

[3] McCrindle BW Rowley AH Newburger JW Burns JC Bolger AF Gewitz M. Diagnosis, treatment, and long-term management of Kawasaki disease: a scientific statement for health professionals from the American Heart Association. Circulation. (2017) 135:e927–99. 10.1161/CIR.0000000000000484 28356445

[4] Newburger JW Takahashi M Burns JC. Kawasaki disease. J Am Coll Cardiol. (2016) 67:1738–49. 10.1016/j.jacc.2015.12.073 27056781

[5] Kato H Koike S Yamamoto M Ito Y Yano E. Coronary aneurysms in infants and young children with acute febrile mucocutaneous lymph node syndrome. J Pediatr. (1975) 86:892–8. 10.1016/S0022-3476(75)80220-4 236368

[6] Fujiwara H Hamashima Y. Pathology of the heart in Kawasaki disease. Pediatrics. (1978) 61:100–7. 263836

[7] Kato H Ichinose E Kawasaki T. Myocardial infarction in Kawasaki disease: clinical analyses in 195 cases. J Pediatr. (1986) 108:923–7. 10.1016/S0022-3476(86)80928-3 3712157

[8] Takahashi K Oharaseki T Yokouchi Y. Update on etio and immunopathogenesis of Kawasaki disease. Curr Opin Rheumatol. (2014) 26:31–6. 10.1097/BOR.0000000000000010 24247115

[9] Menikou S Langford PR Levin M. Kawasaki disease: the role of immune complexes revisited. Front Immunol. (2019) 10:1156. 10.3389/fimmu.2019.01156 31263461 PMC6584825

[10] Sakurai Y. Autoimmune aspects of Kawasaki disease. J Investig Allergol Clin Immunol. (2019) 29:251–61. 10.18176/jiaci.0300 30183655

[11] Rowley AH. Is Kawasaki disease an infectious disorder? Int J Rheum Dis. (2018) 21:20–5. 10.1111/1756-185X.13213 29105346 PMC5777874

[12] Rowley AH Shulman ST. Pathogenesis and management of Kawasaki disease. Expert Rev Anti Infect Ther. (2010) 8:197–203. 10.1586/eri.09.109 20109049 PMC2845298

[13] Furukawa S Matsubara T Motohashi T Sasai K Nakachi S Umezawa Y. Increased expression of Fc epsilon R2/CD23 on peripheral blood B lymphocytes and serum IgE levels in Kawasaki disease. Int Arch Allergy Appl Immunol. (1991) 95:7–12. 10.1159/000235446 1833342

[14] Furukawa S Matsubara T Tsuji K Motohashi T Okumura K Yabuta K. Serum soluble CD4 and CD8 levels in Kawasaki disease. Clin Exp Immunol. (1991) 86:134–9. 10.1111/j.1365-2249.1991.tb05785.x 1914226 PMC1554161

[15] Kuo HC Wang CL Liang CD Yu HR Huang CF Wang L. Association of lower eosinophil-related T helper 2 (Th2) cytokines with coronary artery lesions in Kawasaki disease. Pediatr Allergy Immunol. (2009) 20:266–72. 10.1111/j.1399-3038.2008.00779.x 19438983

[16] Terai M Yasukawa K Honda T Jibiki T Hirano K Sato J. Peripheral blood eosinophilia and eosinophil accumulation in coronary microvessels in acute Kawasaki disease. Pediatr Infect Dis J. (2002) 21:777–81. 10.1097/00006454-200208000-00015 12192168

[17] Xie X Shi X Liu M. The roles of genetic factors in Kawasaki disease: a systematic review and meta-analysis of genetic association studies. Pediatr Cardiol. (2018) 39:207–25. 10.1007/s00246-017-1760-0 29098351

[18] Onouchi Y. The genetics of Kawasaki disease. Int J Rheum Dis. (2018) 21:26–30. 10.1111/1756-185X.13218 29152908

[19] Kuo HC Chang WC Yang KD Yu HR Wang CL Ho SC. Kawasaki disease and subsequent risk of allergic diseases: a population-based matched cohort study. BMC Pediatr. (2013) 13:38. 10.1186/1471-2431-13-38 23522327 PMC3614461

[20] Tsai YJ Lin CH Fu LS Fu YC Lin MC Jan SL. The association between Kawasaki disease and allergic diseases, from infancy to school age. Allergy Asthma Proc. (2013) 34:467–72. 10.2500/aap.2013.34.3697 23998245

[21] Hsieh CY Su CC Shao SC Sung SF Lin SJ Kao Yang YH. Taiwan's national health insurance research database: past and future. Clin Epidemiol. (2019) 11:349–58. 10.2147/CLEP.S196293 31118821 PMC6509937

[22] Lin MC Lai MS. Pediatricians' role in caring for preschool children in Taiwan under the national health insurance program. J Formos Med Assoc. (2009) 108:849–55. 10.1016/S0929-6646(09)60416-2 19933028

[23] Lin LY Warren-Gash C Smeeth L Chen PC. Data resource profile: the national health insurance research database (NHIRD). Epidemiol Health. (2018) 40:e2018062. 10.4178/epih.e2018062 30727703 PMC6367203

[24] Nagata S. Causes of Kawasaki disease-from past to present. Front Pediatr. (2019) 7:18. 10.3389/fped.2019.00018 30805322 PMC6371652

[25] Hara T Nakashima Y Sakai Y Nishio H Motomura Y Yamasaki S. Kawasaki disease: a matter of innate immunity. Clin Exp Immunol. (2016) 186:134–43. 10.1111/cei.12832 27342882 PMC5054572

[26] Fujita Y Nakamura Y Sakata K Hara N Kobayashi M Nagai M. Kawasaki disease in families. Pediatrics. (1989) 84:666–9. 2780128

[27] Uehara R Yashiro M Nakamura Y Yanagawa H. Kawasaki disease in parents and children. Acta Paediatrica (Oslo, Norway: 1992). (2003) 92:694–7. 10.1111/j.1651-2227.2003.tb00602.x 12856980

[28] Abe J Ebata R Jibiki T Yasukawa K Saito H Terai M. Elevated granulocyte colony-stimulating factor levels predict treatment failure in patients with Kawasaki disease. J Allergy Clin Immunol. (2008) 122:1008–13.e8. 10.1016/j.jaci.2008.09.011 18930517

[29] Kuo HC Yang KD Chang WC Ger LP Hsieh KS. Kawasaki disease: an update on diagnosis and treatment. Pediatr Neonatol. (2012) 53:4–11. 10.1016/j.pedneo.2011.11.003 22348488

[30] Lin IC Kuo HC Lin YJ Wang FS Wang L Huang SC. Augmented TLR2 expression on monocytes in both human Kawasaki disease and a mouse model of coronary arteritis. PLoS ONE. (2012) 7:e38635. 10.1371/journal.pone.0038635 22737215 PMC3380902

[31] Wei CC Lin CL Kao CH Liao YH Shen TC Tsai JD. Increased risk of Kawasaki disease in children with common allergic diseases. Ann Epidemiol. (2014) 24:340–3. 10.1016/j.annepidem.2014.02.003 24613197

[32] Webster RJ Carter KW Warrington NM Loh AM Zaloumis S Kuijpers TW. Hospitalisation with infection, asthma and allergy in Kawasaki disease patients and their families: genealogical analysis using linked population data. PLoS ONE. (2011) 6:e28004. 10.1371/journal.pone.0028004 22140498 PMC3225371

[33] Rudzki E Samochocki Z Litewska D Rebandel P Saciuk E Raczka A. Clinical features of atopic dermatitis and a family history of atopy. Allergy. (1991) 46:125–8. 10.1111/j.1398-9995.1991.tb00555.x 2039079

[34] Woon PY Chang WC Liang CC Hsu CH Klahan S Huang YH. Increased risk of atopic dermatitis in preschool children with kawasaki disease: a population-based study in taiwan. Evid Based Complement Alternat Med. (2013) 2013:605123. 10.1155/2013/605123 24069052 PMC3771473

[35] Zhou Y Li X Wang G Li X. Association of FAM167A-BLK rs2736340 polymorphism with susceptibility to Autoimmune diseases: a meta-analysis. Immunol Investig. (2016) 45:336–48. 10.3109/08820139.2016.1157812 27105348

